# The Elastin Receptor Complex: An Emerging Therapeutic Target Against Age-Related Vascular Diseases

**DOI:** 10.3389/fendo.2022.815356

**Published:** 2022-02-11

**Authors:** Dignê Tembely, Aubéri Henry, Laetitia Vanalderwiert, Kevin Toussaint, Amar Bennasroune, Sébastien Blaise, Hervé Sartelet, Stéphane Jaisson, Céline Galés, Laurent Martiny, Laurent Duca, Béatrice Romier-Crouzet, Pascal Maurice

**Affiliations:** ^1^ UMR CNRS 7369 Matrice Extracellulaire et Dynamique Cellulaire (MEDyC), Université de Reims Champagne Ardenne (URCA), UFR Sciences Exactes et Naturelles, Reims, France; ^2^ Institut des Maladies Métaboliques et Cardiovasculaires, INSERM U1048, Université de Toulouse, Toulouse, France

**Keywords:** extracellular matrix, elastin, receptor, neuraminidase, desialylation, signaling, vascular remodeling, aging

## Abstract

The incidence of cardiovascular diseases is increasing worldwide with the growing aging of the population. Biological aging has major influence on the vascular tree and is associated with critical changes in the morphology and function of the arterial wall together with an extensive remodeling of the vascular extracellular matrix. Elastic fibers fragmentation and release of elastin degradation products, also known as elastin-derived peptides (EDPs), are typical hallmarks of aged conduit arteries. Along with the direct consequences of elastin fragmentation on the mechanical properties of arteries, the release of EDPs has been shown to modulate the development and/or progression of diverse vascular and metabolic diseases including atherosclerosis, thrombosis, type 2 diabetes and nonalcoholic steatohepatitis. Most of the biological effects mediated by these bioactive peptides are due to a peculiar membrane receptor called elastin receptor complex (ERC). This heterotrimeric receptor contains a peripheral protein called elastin-binding protein, the protective protein/cathepsin A, and a transmembrane sialidase, the neuraminidase-1 (NEU1). In this review, after an introductive part on the consequences of aging on the vasculature and the release of EDPs, we describe the composition of the ERC, the signaling pathways triggered by this receptor, and the current pharmacological strategies targeting ERC activation. Finally, we present and discuss new regulatory functions that have emerged over the last few years for the ERC through desialylation of membrane glycoproteins by NEU1, and its potential implication in receptor transactivation.

## Introduction

Over the last century, progress in living conditions, public health and medicine have led to a drastic increase in life expectancy worldwide. For the first time, in 2018, the number of people older than 65 years has exceeded the number of children under age of 5, and by 2050, older persons will outnumber adolescents and youth (ages 15 to 24) (1, 2). The explosion of this population part suggests that elderly will play a major role in societies and economies in the coming years, meaning challenges in terms of public health, personal assistance and medical research. This demographic milestone will be accompanied by a major increase in age-associated diseases, such as neurodegenerative diseases, cancer and cardiovascular diseases (3), which essentially double in incidence every 5 years after 60 years old. A major explanation is the progressive and imperceptible vulnerability of the organism to genetic and environmental factors. The degeneration that all organs undergoes with age is the result of a slow and insidious failure of capacities to preserve homeostasis under physiological stress conditions (4). This progressive degenerative state leading to organ fragility is associated with tissue inflammation, stem cells depletion, cellular senescence, extracellular matrix (ECM) alterations, and metabolic dysfunctions (5). These tissue and cellular changes are the visible part of the iceberg, but reflect underlying molecular aberrations in mitochondria, proteostasis, intercellular communication, nutrient uptake, genetic and epigenetic changes (6).

Aging is accompanied by changes in vascular structure and function, especially in the large arteries. Due to their elasticity and resilience capacities, the concentric elastic lamellae of the aorta play a pivotal role in reducing the high systolic pressure at the outlet the heart. In other words, elastic lamellae stretch during cardiac ejection phases allowing the radius of the aorta to increase and to convert the pulsatile flow leaving the heart into a continuous flow in arteries (7). With age, these elastic lamellae exhibit wear characterized by zones of rupture. This leads to loss of elasticity and progressive hardening of the aorta and release of elastin-derived peptides (EDPs) in the circulating blood. These events are accentuated by age-related inflammatory processes and increased activity of elastases such as metalloproteinases (MMP-2, -7, -9, -12), cathepsins, and neutrophil elastase (8). Numerous studies have shown that EDPs are markers of vascular aging and exhibit important biological functions by contributing to progression of cancer (9–11), metabolic (12–14) and cardiovascular diseases (15, 16). These bioactive EDPs, also called elastokines, are well conserved between species and exhibit a xGxxPG consensus sequence (where x represents any amino acid) organized into a type VIII beta-turn structure allowing binding to the elastin-binding protein (EBP) subunit of the elastin receptor complex (ERC) (17). Different membrane receptors can bind tropoelastin, the precursor molecule of elastin, and EDPs, such as galectin-3 (18), the α_v_β_3_ and α_v_β_5_ integrins (19, 20) and a lactose insensitive receptor (21). However, most of the pathophysiological effects reported so far for the elastokines have been attributed to the ERC (8, 22–24).

This review provides an overview of the current state of research on the ERC. After describing the composition of this peculiar receptor, its signaling pathways and the current pharmacological strategies targeting ERC activation, we highlight ERC emerging regulatory functions through desialylation of membrane glycoproteins by its neuraminidase-1 (NEU1) subunit and evoke its potential implication in receptor transactivation.

## Composition of the Elastin Receptor Complex

**Figure 1:**
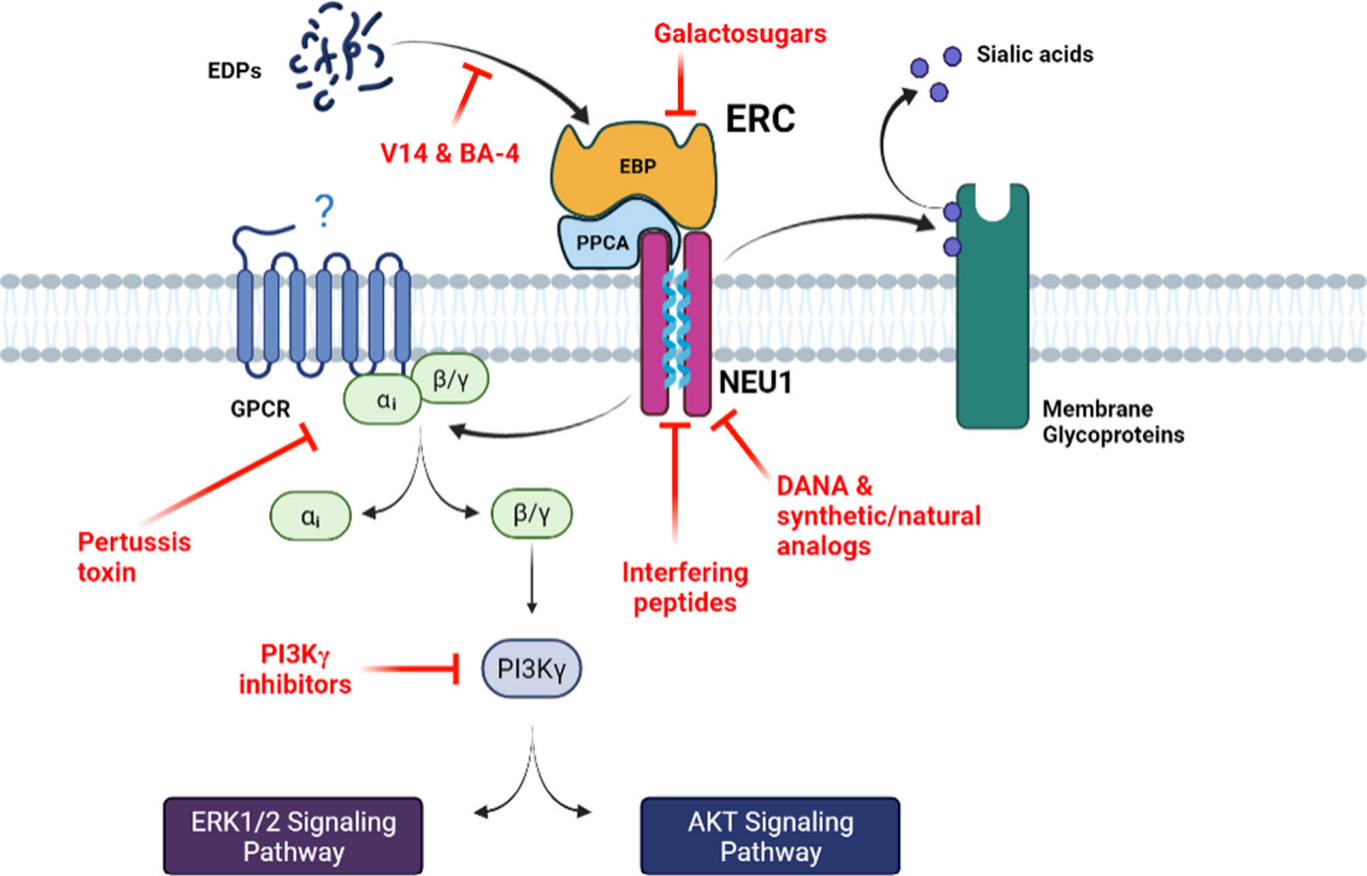
The main signaling pathways mediated by the ERC and associated pharmacological inhibition strategies. Bioactive EDPs binding to the ERC triggers signaling pathways that involve G_αi_ proteins and culminate to ERK1/2 and Akt activation. Increase in NEU1 sialidase activity following elastokines binding to the ERC can also modulate the sialylation level of membrane receptors at its vicinity. The different pharmacological strategies that block ERC activation and signaling, by either blocking the interaction between the elastokines and EBP, inducing EBP shedding or inhibiting NEU1 sialidase activity, are depicted. *DANA, 2-deoxy-2,3-didehydro-N-acetylneuraminic acid; EBP, elastin-binding protein; EDPs, elastin-derived peptides; ERC, elastin receptor complex; GPCR, G-protein coupled receptor; NEU1, neuraminidase-1; PPCA, protective protein/cathepsin A*.

The ERC is a heterotrimeric receptor containing a peripheral protein of 67 kDa called EBP, the protective protein/cathepsin A (PPCA, 55 kDa) and the transmembrane NEU1 (61 kDa) (25) (**Figure 1**). The ERC has a strong homology with the lysosomal β-galactosidase (β-gal) complex involved in the degradation of glycoconjugates wherein EBP is replaced by β-gal. Actually, EBP is a spliced variant of β-gal resulting from the deletion of 3 of the 16 exons encoding the β-gal protein and two frameshifts (26, 27). This splicing results in replacement of a 162-residue portion of the catalytic domain by a 32-residue sequence unique for EBP (28) that defines a binding pocket for peptides and proteins containing xGxxPG motifs, such as the elastokines, tropoelastin, and several other matrix proteins (29). This spliced version of β-gal is devoid of enzymatic activity but kept galactolectin properties and binds β-galactosugars such as galactose and lactose. Binding of β-galactosugars to EBP plays a pivotal role during elastic fibers formation by regulating tropoelastin molecules release from EBP for subsequent assembly into the growing elastic fiber. Indeed, binding of galactosugars to the lectin domain of EBP causes conformational changes in the protein, leading to its dissociation from tropoelastin and other components of the cell surface-immobilized complex (30, 31) and subsequent coordinated anchoring of tropoelastin molecules to fibrillar glycoproteins that constitute the surrounding fibrillar mantle of elastic fibers. 

Lysosomal PPCA is a serine carboxypeptidase that acts as a chaperone and protective protein by helping intracellular routing, lysosomal localization and activation of NEU1 (32, 33), and β-gal stabilization in lysosomes (34, 35). Besides its protective function, PPCA has a cathepsin A-like enzymatic activity at acid pH and a deamidase/esterase activity at neutral pH (36). Within the ERC, PPCA has similar protective function by maintaining EBP integrity (37) and, in contrast to NEU1, the catalytic activity of PPCA is not required for signal transduction through the ERC (38).

NEU1 is part of the mammalian sialidase family that are exoglycosidases removing terminal sialic acid residues from glycoproteins, glycolipids and oligosaccharides in lysosomes. NEU1 essentially catalyzes the hydrolytic cleavage of terminal sialic acid residues from oligosaccharides and glycoproteins (39). In addition to be expressed in lysosomes, NEU1 is also present at the plasma membrane where it regulates a myriad of membrane glycoproteins by desialylation, such as integrins (40), receptor tyrosine kinases (RTKs) (12, 41, 42), Toll-like receptors (TLRs) (43, 44), and platelet GPIb (45), resulting in modulation of receptor activation and signaling. Within the ERC, the catalytic activity of NEU1 plays a key role for signal transduction through this receptor (38, 46) and constitutes the catalytic subunit of the ERC. How binding of elastokines to the ERC induces increase in sialidase activity of NEU1 within the heterotrimeric complex is still unknown. So far, the crystallographic structure of human NEU1 is not resolved and all the proposed structural models for NEU1 are homology models based on the crystal structure of the cytosolic human NEU2 (47, 48). By combining biology and biochemistry together with structural biophysics and computational approaches, we demonstrated that human NEU1 is present as dimers at the plasma membrane (49). Two potential transmembrane domains were identified and the corresponding peptides were prone to form stable α-helices in membrane-mimicking environments. Importantly, the 316-333 domain of NEU1 was suited for self-association, and *in vitro* experiments further confirmed the ability of membrane NEU1 to dimerize. Introduction of point mutations within this dimerization interface was associated with substantial disruption of membrane NEU1 dimerization and decrease of membrane sialidase activity (49). From these original results, it was proposed that membrane dimerization of NEU1 controls its catalytic activity.

Due to the lack of structural data for NEU1 and EBP, the composition and structure of the ERC is still unknown. The ERC is classically depicted as a complex containing one copy of EBP, one copy of PPCA and one copy of NEU1 dimer. However, a recent study has revealed the first structural model of the lysosomal multienzyme complex core by cryo-electron microscopy, composed of β-gal and PPCA recombinantly expressed in insect cells (50). This 0.8 MDa complex is composed of three β-gal dimers and three PPCA dimers, adopting a triangular architecture maintained through six copies of a unique β-gal-PPCA polar interface. Whether this model could apply for the ERC remains to be determined. As mentioned above, the β-gal splicing to EBP results in replacement of a 162-residue portion by a 32-amino acid sequence unique to EBP. In the proposed model, the β-gal residues involved in the β-gal-PPCA interface are almost all lacking in EBP (50). Therefore, the structure of the ERC is likely quite different. Further studies are needed to understand the interplay between these three enzymes in lysosomes and at the cell surface.

## The Elastin Receptor Complex and Its Signaling Pathways

Elastokines are able to modulate a large number of cellular processes including chemotaxis (51, 52), proliferation (53–56), protease synthesis (11, 57–59), ion influx (60, 61), migration (59) and invasion (18, 62) for a significant number of normal and tumor cells. Furthermore, elastokines modulate the inflammatory response (63, 64) and are involved in the development and/or progression of many pathologies such as cancer (23), diabetes (12), nonalcoholic steatohepatitis (14), atherosclerosis (15), and modulation of arterial thrombosis (16). Elastokines also exhibit beneficial effects such as in cardioprotection (65), tissue remodeling and wound healing (66, 67).

One of the first signaling pathways identified for the ERC came from the pioneer study of Varga et al. showing that elastokines are able to stimulate the oxidative burst, IP3 production and intracellular free Ca^2+^ mobilization in human monocytes and polymorphonuclear leukocytes through a pertussis toxin (PTX)-sensitive G_αi/o_ protein (68) (**Figure 1**). Mochizuki et al. then confirmed the involvement of G_αi/o_ proteins in ERC-mediated signaling pathways in arterial smooth muscle cells (55). They further showed that elastokines binding to the ERC causes opening of L-type Ca^2+^ channels and Ca^2+^ entry into the cytosol, leading to a sequence of tyrosine phosphorylations involving FAK, c-Src, platelet-derived growth factor receptor kinase and the Ras-Raf-MEK1/2-ERK1/2 pathways. These phosphorylation events lead to an increased proliferation of arterial smooth muscle cells and cytoskeleton reorganization. In the same time, Duca et al. demonstrated that the MEK-ERK1/2 cascade is also activated by elastokines in human skin fibroblasts leading to increased production of AP-1 transcription factor and pro-MMP1 (69). They also highlighted two different pathways that can lead to activation of MEK-ERK1/2, the first one involving increased production of cAMP and activation of PKA, and the second one acting through the activation of PI3K. It was shown later that the PI3K involved in ERC signaling is the PI3Kγ isoform that is activated through the βγ subunits of a PTX sensitive heterotrimeric G_i/o_ protein (70).

Involvement of NEU1 catalytic activity in ERC signaling pathways was reported for the first time by Duca et al. in 2007 (38). They showed that ERK1/2 activation and pro-MMP1 production in response to elastokines binding to the ERC depend on NEU1 sialidase activity. Indeed, the use of NEU1 catalytically inactive mutant and NEU1 siRNA was shown to abolish elastokines effects. Interestingly, they also demonstrated that direct stimulation of cells by exogenous sialic acid (*N*-acetyl-α-D-neuraminic acid, Neu5Ac) mimics elastokines effects, indicating that the enzymatic (sialidase) activity of the NEU1 subunit of the ERC is responsible for its signal transduction, presumably through desialylation and sialic acid generation. NEU1 can cleave sialic acids from different substrates such as glycoproteins, oligosaccharides and glycolipids at the α-2,6 and/or α-2,3 glycan-linkages. In human skin fibroblasts, Rusciani et al. have identified the GM3 ganglioside as one of the NEU1 substrates (46). They showed that stimulation of cells by elastokines induced GM3 desialylation and production of lactosylceramide (LacCer) and that these events were blocked by lactose (EBP antagonist) and NEU1 siRNA. As for Neu5Ac, LacCer also reproduced elastokines stimulating effects on ERK1/2 phosphorylation. Similar observations were recently reported in the pre-adipocyte 3T3-L1 cell line (13). Taken together, these findings strongly suggest that sialic acid plays by itself an important role in ERC-mediated signaling pathways. Whether sialic acids can directly generate intracellular signaling or act through other membrane receptors, such as members of the sialic acid-binding immunoglobulin-like lectin (Siglec) family remains to be determined. In addition to its involvement in sialic acid generation, and as described below, NEU1 also plays a pivotal role in ERC-mediated signaling pathways and biological effects through desialylation of membrane glycoproteins.

## The Current Pharmacological Strategies Targeting ERC Activation

Different strategies are available to target ERC activation and related signaling pathways that either block elastokines binding to the ERC, induce shedding of EBP from the receptor complex and ERC inactivation, or inhibit NEU1 catalytic activity (**Figure 1**). Blocking the interaction between the elastokines and the EBP subunit of the ERC can be achieved by using the BA-4 monoclonal antibody. This blocking antibody binds to insoluble elastin, tropoelastin and to EDPs (71). BA-4 binds to xGxxPG motifs and thereby is used as blocker of the interaction between elastokines and the EBP subunit of the ERC. This approach has been used in several studies and was shown to prevent elastin damage and to neutralize ERC-mediated deleterious effects in emphysema, abdominal aortic aneurysms and aortic disease associated to Marfan syndrome in mice (72–76). Binding of elastokines to the ERC can also be blocked by the V14 peptide (VVGSPSAQDEASPL), a 14mer peptide corresponding to part of the elastin binding sequence of EBP that binds xGxxPG motifs found in elastokines (29). This strategy has been also widely used in the literature for *in vitro* and *in vivo* applications (11, 59, 65, 77, 78). ERC activation can also be inhibited by galactosugars. As mentioned above, EBP is a spliced version of β-gal that lost enzymatic activity but kept galactolectin properties. Accordingly, it was demonstrated that EBP can be eluted from elastin affinity column but also released from the cell surface by galactosugars (30, 31). The use of these compounds, mainly lactose and chondroitin sulfate, results in shedding of EBP from the ERC and inhibits ERC-mediated signaling pathways. Therefore, galactosugars are commonly used as antagonists of the ERC (11, 12, 14, 38, 57, 63, 65, 70, 78, 79).

Another available strategy to inhibit ERC-mediated signaling pathways is based on the blockade on NEU1 activation following elastokines binding to the receptor. Due to the lack of selective inhibitors, the majority of studies dealing with human neuraminidases have used the 2-deoxy-2,3-didehydro-*N-*acetylneuraminic acid (DANA), a nonselective inhibitor of the four neuraminidase isoenzymes, or viral sialidase inhibitors such as oseltamivir phosphate or zanamivir. Although viral sialidase inhibitors have also broad specificity for bacterial neuraminidases, studies that have assessed the activity of zanamivir and oseltamivir phosphate against human neuraminidases have reported weaker efficacy (80, 81) and contradictory results. Analysis of DANA binding to the viral neuraminidase active site by X-ray crystallography (82, 83) shows identical interactions as the natural substrate, sialic acid (84). Despite the fact that human neuraminidase isoforms share a high-level amino acid conservation in the active site and its vicinity, they have some striking differences, particularly in the DANA’s glycerol binding group, which has been exploited to design selective inhibitors. In this context, C9-amide derivatives of DANA were the first DANA analogs investigated. Among them, the C9-butyl-*N*-amide derivative (C9-BA-DANA) was shown to be 200-fold more selective (IC_50_ 10µM) for human NEU1 over NEU2, NEU3, and NEU4 with respect to DANA (85). Thereafter, another group has reported DANA analogs at C5 and C9 positions and the best NEU1 inhibitor identified was the C5-hexanamido-C9-acetamido analogue with a *K*_i_ of 53 ± 5 nM and a selectivity increased by 340-fold over the other isoenzymes (86). Both DANA analogs have been successfully validated in *in vitro* and *in vivo* studies (87, 88). As described above, we previously identified two segments in human NEU1 as potential transmembrane (TM) domains. Among them, the 316-333 domain (referred as TM2) was shown to form a dimerization interface that controls NEU1 catalytic activity (49). From these results, we developed an original strategy based on the use of interfering peptides to target this dimerization interface. We showed that these interfering peptides were able to interact with NEU1 TM2, to disrupt membrane NEU1 dimerization and to inhibit its sialidase activity (89). *In vivo* application of these new promising selective NEU1 inhibitors is currently under investigation. Finally, recent works have been done to identify neuraminidase inhibitors from natural bioactive molecules extracted from plants (90–92). These natural bioactive compounds could be used as a starting point for the development of new natural putative NEU1 inhibitors and the design of more potent synthetic compounds. In this context, we recently reported the purification of three natural compounds from the leaves of the *Olyra latifolia* plant that present structural analogy with DANA and possess inhibitory effects against human NEU1 (90).

Finally, another valuable strategy to block ERC activation relies on the inhibition of key proteins involved in ERC-mediated signal transduction. As mentioned above, one pivotal element is the G_αi/o_ protein as inactivation of G_i/o_ proteins by PTX strongly inhibits ERC-mediated signaling pathways (55, 68, 70). Another signaling relay is PI3Kγ. Blocking PI3Kγ inhibits ERC-mediated signaling pathways (70) and PI3Kγ deficiency in mice strongly reduces EDP-induced reactive oxygen species (ROS) production and migration of monocytes and proatherogenic effects of elastokines in a mouse model of atherosclerosis (15). However, G_i/o_ proteins and PI3Kγ being common transducers for a multitude of receptors, blocking G_i/o_ proteins or PI3Kγ may not be a viable therapeutical option to inhibit ERC activation.

## Emerging Regulatory Functions for the ERC Through Desialylation by NEU1

### Regulation of Membrane Receptor Functions Through NEU1 Desialylation

**Figure 2:**
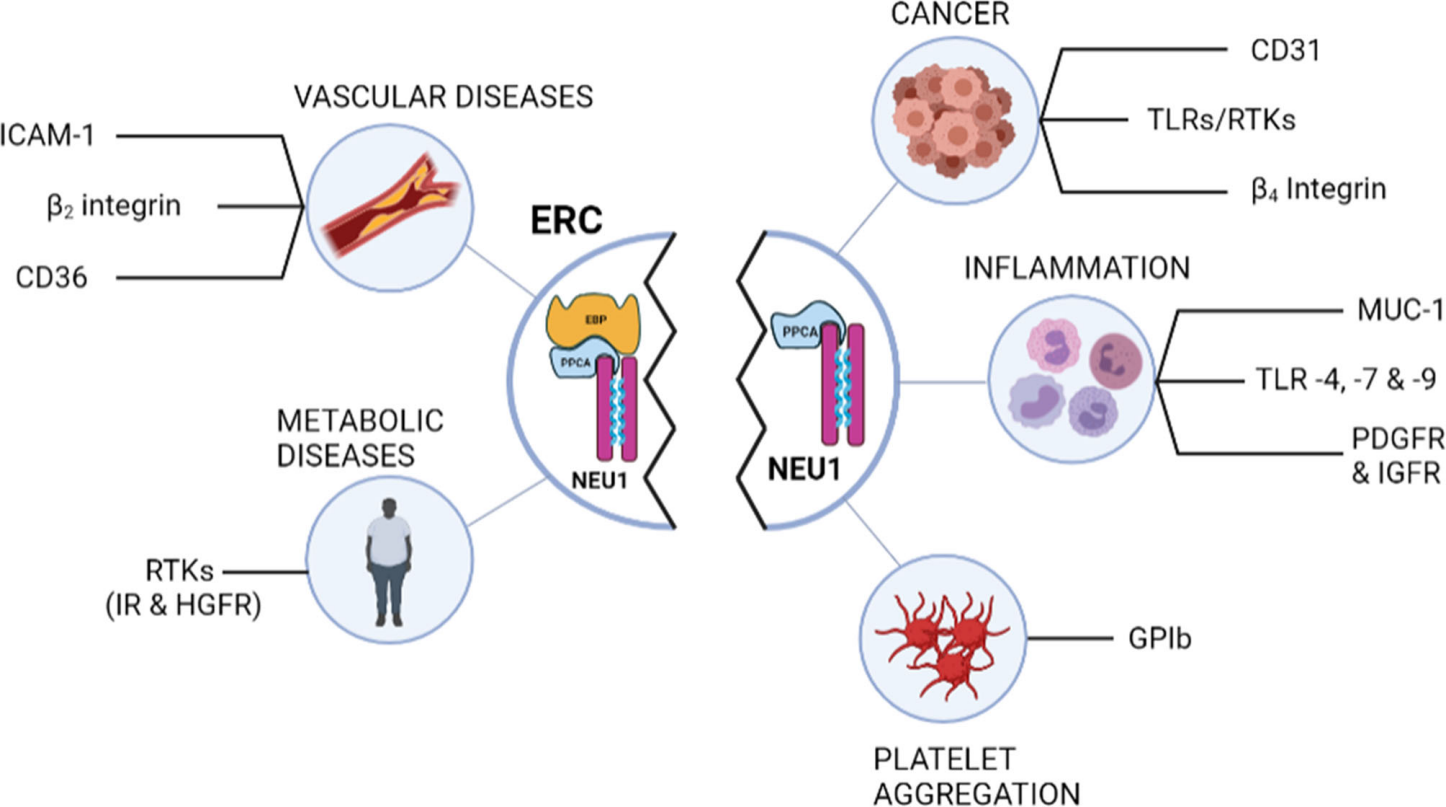
Schematic representation of the membrane glycoproteins regulated by desialylation through NEU1 and potential biological relevance in various diseases. Left panel shows the membrane glycoproteins regulated by desialylation through NEU1 after ERC involvement. Right panel lists the other membrane glycoproteins that have been shown to be desialylated by NEU1. Whether these latter can be modulated by the ERC remains to be evaluated. *EBP, elastin-binding protein; ERC, elastin receptor complex; HGFR, hepatic growth factor receptor; ICAM-1, intercellular adhesion molecule-*1; *IR, insulin receptor; MUC-1, mucine-1; NEU1, neuraminidase-1; PDGFR, Platelet-derived growth factor* receptor; *PPCA, protective protein/cathepsin A; IGFR, insulin-like growth factor receptor; RTK, receptor tyrosine kinase; TLR, toll-like receptor*.

Along with the pivotal role of NEU1 for signal transduction by the ERC, accumulative data from the last few years highlighted that the binding of elastokines to the ERC could also modulate membrane receptor functions at the vicinity of the ERC by desialylation through NEU1, opening new regulatory effects for the ERC (**Figure 2**). For instance, Blaise et al. have reported that chronic administration of EDPs in mice promote insulin resistance through modulation of the insulin receptor (IR) by the ERC (12). By analyzing mouse tissues, they showed that elastokines stimulation led to the interaction between NEU1 and IR, IR desialylation and decrease of IR, Akt and Foxo-1 phosphorylation. These modulatory effects of elastokines were reversed by the sialidase inhibitor DANA and ERC antagonists such as chondroitin sulfate (12). By their ability to increase membrane NEU1 sialidase activity, elastokines binding to the ERC was also shown to be able to regulate the signaling pathways of other RTKs, such as the hepatic growth factor receptor (HGFR), also known as C-MET (14). Indeed, Blaise et al. have reported that chronic accumulation of EDPs in mice led to non-alcoholic steatohepatitis through a mechanism involving desialylation of HGFR and inhibition of the LKB1/AMPK phosphorylation cascade (14).

A similar mode of action was highlighted by Kawecki et al. for another family of receptors, the class B scavenger CD36 receptor (78). In the search for new interaction partners of membrane NEU1, they developed a proteomic approach and identified CD36 as a new interaction partner of NEU1. Using human macrophages differentiated from the THP-1 cell line, and after validation of the constitutive interaction between NEU1 and CD36, they reported that elastokines binding to the ERC induced desialylation of CD36 and potentiation of oxidized LDL uptake by macrophages (78). Finally, unpublished data from our group revealed that this mode of action also applies for the β_2_ integrin in monocytes and for the intercellular adhesion molecule-1 (ICAM-1) in endothelial cells. By stimulating the catalytic activity of NEU1, elastokines binding to the ERC induces desialylation of both monocyte β_2_ integrin and endothelial ICAM-1 through NEU1, and enhances monocyte adhesion to endothelial cells and monocyte transendothelial migration. Thus, by this newly discovered mode of action, new biological functions are anticipated for NEU1 through the ERC in diseases involving elastic fibers remodeling and degradation, and opens new avenues in the fine-tuning of membrane receptor activation and signaling. Importantly, a large amount of other membrane glycoproteins has been shown to be modulated by desialylation through NEU1 such as the β_4_ integrin (40), CD31 (93), platelet GPIb (45), TLR4 (43) and several RTKs including TrkA (41), IGF, PDGF and EGF receptors (42, 94) and MUC1 (42) (**Figure 2**). Whether these membrane receptors can be regulated by desialylation through ERC involvement remains to be shown but anticipates new regulatory functions to be discovered for elastokines and the ERC.

### Potential Involvement of the Elastin Receptor Complex in Receptor Transactivation

Transactivation of RTKs by G-protein coupled receptors (GPCRs), and reciprocally, is a well-characterized phenomenon and has been extensively reviewed elsewhere (95–97). Receptor transactivation has been shown to play important roles in various physiological and pathological processes such as in cancer and cardiovascular diseases, thereby providing new insights and new potential targets. RTKs can be activated by GPCRs in a ligand-dependent or -independent manner. Ligand-dependent transactivation mainly occurs *via* MMPs or a disintegrin and metalloproteinases (ADAMs) upon GPCR activation. Activated MMPs or ADAMs then cleave membrane-bound RTK pro-ligands that bind to RTKs and trigger downstream signaling. Transactivation can also occur through ligand-independent mechanisms. Effector proteins activated following GPCR activation, such as Src, PKC and Pyk, can directly activate RTKs *via* phosphorylation of their C terminus extremities. In addition, secondary messenger molecules such as ROS can also mediate direct activation of RTKs (96).

Transactivation of RTKs by GPCRs is not unidirectional as a large body of evidence indicates that RTKs can also transactivate GPCRs in a ligand-dependent or -independent manner. Ligand-dependent transactivation of GPCRs by RTKs results from the synthesis and secretion of the ligand of the transactivated GPCR, which binds and activates the GPCR in an autocrine and/or paracrine manner. For instance, in human breast carcinoma cells, IGF-1 can transactivate the G protein-coupled chemokine receptor CCR5 through enhancement of synthesis and secretion of RANTES mRNA, the natural ligand of CCR5 (98). Ligand-independent transactivation of GPCRs by RTKs rather involves formation of GPCR-RTK complexes and sometimes phosphorylation of the transactivated GPCRs (99). Comparable crosstalks have been proposed for GPCRs and Toll-like receptors (TLRs) (100).

Interesting findings from the last decade came from the group of Szewczuk that describes a novel organizational signaling platform wherein NEU1 is placed at the center of tripartite molecular complexes involving GPCRs, the matrix metalloproteinase 9 (MMP-9) and RTKs or TLRs (43, 101–105), opening new roles for NEU1, and potentially for the ERC, in receptor crosstalk and transactivation. It was uncovered that binding of GPCR agonists to their cognate receptor induces GPCR-signaling processes *via* Gα_i_ proteins and subsequent MMP-9 activation leading to increase of NEU1 sialidase activity. In turn, the sialidase activity of NEU1 tethered to the RTK or TLR hydrolyzes the α-2,3-sialyl residues of the receptor, enabling removal of steric hindrance for receptor association and subsequent RTK or TLR activation. This was illustrated in the human IR-expressing rat hepatoma cell line where GPCR agonists such as bombesin, bradykinin, angiotensin I and angiotensin II, were shown to dose-dependently induce NEU1 sialidase activity and IR signaling in the complete absence of insulin. Among these GPCR agonists, angiotensin II was found to be the most potent inducer of IRβ and insulin receptor substrate-1 (IRS-1) phosphorylation. Furthermore, these effects were blocked by the sialidase inhibitor oseltamivir phosphate and the neuromedin B GPCR (NMBR) inhibitor BIM-23127 (106). These findings were consistent with a previous report describing the regulatory role of NMBR in inducing NEU1 sialidase activity and MMP-9 crosstalk required for IRβ desialylation and receptor activation (104). Together with a prior study showing that the same GPCR agonists induced NEU1 sialidase activity on the cell surface of primary bone marrow macrophages, resulting in NEU1-mediated desialylation, dimerization, and transactivation of TLR4 in the absence of natural ligand (102), these findings support a central role for NEU1 in receptor transactivation processes.

An intriguing issue that remains to be resolved for this model is the link between MMP-9 and NEU1 and how MMP-9 proceeds for NEU1 activation. It is assumed that GPCR need to be tethered to the RTK or TLR in order to activate MMP-9 already in complex with the ERC containing NEU1. In turn, the metallo-elastase activity of MMP-9 would cleave the EBP subunit from the ERC, thereby exposing the catalytic sialidase domain of NEU1 (101). To our knowledge, involvement of MMP-9 for the release of EBP from the ERC has not been demonstrated so far. Rather, and as mentioned previously, dissociation of EBP from the ERC is known to be triggered by binding of galactosugars onto EBP. This process is involved for assembly of tropoelastin molecules onto the microfibrillar scaffold during elastogenesis (25). It is proposed, that by removing terminal sialic acid residues from carbohydrate chains protruding from microfibrillar glycoproteins, NEU1 (linked to EBP and PPCA) causes unmasking of penultimate galactosugars, which in turn interact with the galactolectin site of EBP and induces release of the transported tropoelastin molecule from EBP. In the meantime, EBP dissociates from NEU1 and PPCA and is recycled back to the endosomal compartments. Once in the recycling endosomes, EBP reassociates with NEU1 and PPCA, and binds again new tropoelastin molecules delivered from the endoplasmic reticulum to chaperone them to the cell surface (25). For more details, the reader is referred to major reviews in the field (7, 107, 108). Since binding of elastokines to EBP directly activates NEU1 and increases its sialidase activity, it is tempting to speculate that the ERC may be involved in such receptor crosstalk and transactivation.

## Concluding Remarks and Future Challenges

Vascular aging is associated with an extensive remodeling of the ECM. Over the last decade, elastic fibers fragmentation and release of EDPs have emerged as major contributors of vascular ECM remodeling and associated diseases occurring with aging. The different studies summarized in this review show that the ERC may play a pivotal role in such effects. The new regulatory functions that have emerged over the last few years for the ERC through membrane glycoproteins desialylation by its NEU1 subunit, and the potential implication of the ERC in receptor transactivation, suggest that another biological and regulatory functions remain to be discovered for the ERC. Given the fact that GPCRs form the largest human membrane protein family, including approximately 800 members, and are the target of around 34% of all drugs approved by the US Food and Drug Administration (109), one main issue is likely to assess whether the ERC may form complexes with and modulate GPCR activation. In this context, the use of sensitive approaches dedicated to the identification of membrane interaction partners, such as the membrane yeast two-hybrid (MYTH) screen (110), has to be considered. MYTH is a very sensitive technique that adapts the principle of split-ubiquitin for use as potent *in vivo* sensor of direct protein-protein interactions and is optimized for the detection of large-scale membrane protein interactions. The use of such an approach will definitely help in better understanding the role played by the ERC, through its NEU1 catalytic subunit, in health and diseases, and should open new avenues for pharmacological strategies targeting the ERC and its biological effects. It is tempting to speculate that disrupting the interaction between elastokines and the ERC or blocking the signaling pathways triggered the receptor may represent efficient and selective therapeutical targets in the future. Although pharmacological strategies are already available and currently used in research, a complete structural picture of the complex receptor is still lacking but is absolutely required to open the way to the design of new antagonists targeting the ERC and to prevent the deleterious effects of these ECM-derived peptides. Indeed, the unraveling of the 3D structure of the whole molecular complex is a prerequisite to the understanding of the interaction mechanisms as well as the structural relationships between its three subunits. Among the ERC constitutive proteins, only the crystal structure of the protective protein has been solved (111). For EBP and NEU1, homology models have been released (29, 48). However, NEU1 is, by definition, a lysosomal sialidase and the current homology models, based on the human cytosolic NEU2, cannot account for such membrane localization. As mentioned in this review, another main issue that remains to be investigated is how elastokines binding to the ERC increases NEU1 catalytic activity within the receptor complex. The optimum pH for the lysosomal enzyme is acidic (pH 4.5) whereas the plasma membrane-bound sialidase has an optimum pH at around 6.5. Therefore, this increased membrane NEU1 sialidase activity following cell stimulation by elastokines could not be due to the lysosomal pool of this sialidase (112). Moreover, it has been demonstrated that EBP is never targeted to lysosomes (26–28). Conformational changes within NEU1 are rather favored but remains to be demonstrated.

## Author Contributions

All authors listed have made a substantial, direct, and intellectual contribution to the work, and approved it for publication.

## Funding

This review was supported by grants from ANR (ANR-18-CE44-0017), CNRS and URCA. Authors also thank the Région Grand Est, the Fonds Regional de Coopération pour la Recherche (Grand Est-Alsace Champagne-Ardenne Lorraine) for their grant to the project “Pathological Obesity and Metabolic Aging (OMAGE)” and the Ligue Nationale Contre le Cancer (Projet de Recherches Interrégional). DT received his PhD salary from ANR.

## Conflict of Interest

The authors declare that the research was conducted in the absence of any commercial or financial relationships that could be construed as a potential conflict of interest.

The reviewer, AP, declared a past collaboration with one of the authors, LM, to the handling editor.

## Publisher’s Note

All claims expressed in this article are solely those of the authors and do not necessarily represent those of their affiliated organizations, or those of the publisher, the editors and the reviewers. Any product that may be evaluated in this article, or claim that may be made by its manufacturer, is not guaranteed or endorsed by the publisher.

## References

[1] Nations U. World Population Aging. (2017).

[2] Nations U. Shifting Demographics. (2020).

[3] Melzer D Pilling LC Ferrucci L. The Genetics of Human Ageing. Nat Rev Genet (2020) 21:88–101. doi: 10.1038/s41576-019-0183-6 31690828 PMC9934000

[4] Fulop T Larbi A Witkowski JM McElhaney J Loeb M Mitnitski A. Aging, Frailty and Age-Related Diseases. Biogerontology (2010) 11:547–63. doi: 10.1007/s10522-010-9287-2 20559726

[5] Lopez-Otin C Blasco MA Partridge L Serrano M Kroemer G. the Hallmarks of Aging. Cell (2013) 153:1194–217. doi: 10.1016/j.cell.2013.05.039 PMC3836174 23746838

[6] Chakravarti D LaBella KA DePinho RA. Telomeres: History, Health, and Hallmarks of Aging. Cell (2021) 184:306–22. doi: 10.1016/j.cell.2020.12.028 PMC8081271 33450206

[7] Wahart A Bennasroune A Schmelzer CEH Laffargue M Blaise S Romier-Crouzet B Sartelet H. Role of Elastin and Elastin-Derived Peptides in Arterial Stiffness: From Synthesis to Potential Therapeutic Interventions. In: Chirinos J, editor. Textbook of Arterial Stiffness and Pulsatile Hemodynamics in Health and Disease, 1st Edition. Elsevier Science & Technology (2022).

[8] Duca L Blaise S Romier B Laffargue M Gayral S El Btaouri H. Matrix Ageing and Vascular Impacts: Focus on Elastin Fragmentation. Cardiovasc Res (2016) 110:298–308. doi: 10.1093/cvr/cvw061 27009176

[9] Bretaudeau C Baud S Dupont-Deshorgue A Cousin R Brassart B Brassart-Pasco S. AG-9, an Elastin-Derived Peptide, Increases *In Vitro* Oral Tongue Carcinoma Cell Invasion, Through an Increase in MMP-2 Secretion and MT1-MMP Expression, in a RPSA-Dependent Manner. Biomolecules (2020) 11:39. doi: 10.3390/biom11010039 33396696 PMC7823410

[10] Devy J Duca L Cantarelli B Joseph-Pietras D Scandolera A Rusciani A. Elastin-Derived Peptides Enhance Melanoma Growth *In Vivo* by Upregulating the Activation of Mcol-a (MMP-1) Collagenase. Br J Cancer (2010) 103:1562–70. doi: 10.1038/sj.bjc.6605926 PMC2990576 20959825

[11] Toupance S Brassart B Rabenoelina F Ghoneim C Vallar L Polette M. Elastin-Derived Peptides Increase Invasive Capacities of Lung Cancer Cells by Post-Transcriptional Regulation of MMP-2 and Upa. Clin Exp Metastasis (2012) 29:511–22. doi: 10.1007/s10585-012-9467-3 22434583

[12] Blaise S Romier B Kawecki C Ghirardi M Rabenoelina F Baud S. Elastin-Derived Peptides are New Regulators of Insulin Resistance Development in Mice. Diabetes (2013) 62:3807–16. doi: 10.2337/db13-0508 PMC3806616 23919962

[13] Hocine T Blaise S Hachet C Guillot A Sartelet H Maurice P. Lactosylceramide Induced by Elastin-Derived Peptides Decreases Adipocyte Differentiation. J Physiol Biochem (2020) 76:457–67. doi: 10.1007/s13105-020-00755-z 32592089

[14] Romier B Ivaldi C Sartelet H Heinz A Schmelzer CEH Garnotel R. Production of Elastin-Derived Peptides Contributes to the Development of Nonalcoholic Steatohepatitis. Diabetes (2018) 67:1604–15. doi: 10.2337/db17-0490 29802129

[15] Gayral S Garnotel R Castaing-Berthou A Blaise S Fougerat A Berge E. Elastin-Derived Peptides Potentiate Atherosclerosis Through the Immune Neu1-PI3Kgamma Pathway. Cardiovasc Res (2014) 102:118–27. doi: 10.1093/cvr/cvt336 24357053

[16] Kawecki C Hezard N Bocquet O Poitevin G Rabenoelina F Kauskot A. Elastin-Derived Peptides are New Regulators of Thrombosis. Arterioscler Thromb Vasc Biol (2014) 34:2570–8. doi: 10.1161/ATVBAHA.114.304432 25341794

[17] Brassart B Fuchs P Huet E Alix AJ Wallach J Tamburro AM. Conformational Dependence of Collagenase (Matrix Metalloproteinase-1) Up-Regulation by Elastin Peptides in Cultured Fibroblasts. J Biol Chem (2001) 276:5222–7. doi: 10.1074/jbc.M003642200 11084020

[18] Pocza P Suli-Vargha H Darvas Z Falus A. Locally Generated VGVAPG and VAPG Elastin-Derived Peptides Amplify Melanoma Invasion *via* the Galectin-3 Receptor. Int J Cancer (2008) 122:1972–80. doi: 10.1002/ijc.23296 18076073

[19] Bax DV Rodgers UR Bilek MM Weiss AS. Cell Adhesion to Tropoelastin is Mediated *via* the C-Terminal GRKRK Motif and Integrin Alphavbeta3. J Biol Chem (2009) 284:28616–23. doi: 10.1074/jbc.M109.017525 PMC2781405 19617625

[20] Lee P Bax DV Bilek MM Weiss AS. A Novel Cell Adhesion Region in Tropoelastin Mediates Attachment to Integrin Alphavbeta5. J Biol Chem (2014) 289:1467–77. doi: 10.1074/jbc.M113.518381 PMC3894329 24293364

[21] Maeda I Mizoiri N Briones MP Okamoto K. Induction of Macrophage Migration Through Lactose-Insensitive Receptor by Elastin-Derived Nonapeptides and Their Analog. J Pept Sci (2007) 13:263–8. doi: 10.1002/psc.845 17394124

[22] Bennasroune A Romier-Crouzet B Blaise S Laffargue M Efremov RG Martiny L. Elastic Fibers and Elastin Receptor Complex: Neuraminidase-1 Takes the Center Stage. Matrix Biol (2019) 84:57–67. doi: 10.1016/j.matbio.2019.06.007 31226402

[23] Scandolera A Odoul L Salesse S Guillot A Blaise S Kawecki C. The Elastin Receptor Complex: A Unique Matricellular Receptor With High Anti-Tumoral Potential. Front Pharmacol (2016) 7:32. doi: 10.3389/fphar.2016.00032 26973522 PMC4777733

[24] Wahart A Hocine T Albrecht C Henry A Sarazin T Martiny L. Role of Elastin Peptides and Elastin Receptor Complex in Metabolic and Cardiovascular Diseases. FEBS J (2019) 286:2980–93. doi: 10.1111/febs.14836 30946528

[25] Hinek A Pshezhetsky AV von Itzstein M Starcher B. Lysosomal Sialidase (Neuraminidase-1) Is Targeted to the Cell Surface in a Multiprotein Complex That Facilitates Elastic Fiber Assembly. J Biol Chem (2006) 281:3698–710. doi: 10.1074/jbc.M508736200 16314420

[26] Hinek A Rabinovitch M Keeley F Okamura-Oho Y Callahan J. The 67-Kd Elastin/Laminin-Binding Protein is Related to an Enzymatically Inactive, Alternatively Spliced Form of Beta-Galactosidase. J Clin Invest (1993) 91:1198–205. doi: 10.1172/JCI116280 PMC288077 8383699

[27] Privitera S Prody CA Callahan JW Hinek A. The 67-Kda Enzymatically Inactive Alternatively Spliced Variant of Beta-Galactosidase is Identical to the Elastin/Laminin-Binding Protein. J Biol Chem (1998) 273:6319–26. doi: 10.1074/jbc.273.11.6319 9497360

[28] Morreau H Galjart NJ Gillemans N Willemsen R van der Horst GT d’Azzo A. Alternative Splicing of Beta-Galactosidase Mrna Generates the Classic Lysosomal Enzyme and a Beta-Galactosidase-Related Protein. J Biol Chem (1989) 264:20655–63. doi: 10.1016/S0021-9258(19)47114-7 2511208

[29] Blanchevoye C Floquet N Scandolera A Baud S Maurice P Bocquet O. Interaction Between the Elastin Peptide VGVAPG and Human Elastin Binding Protein. J Biol Chem (2013) 288:1317–28. doi: 10.1074/jbc.M112.419929 PMC3543015 23166321

[30] Hinek A Wrenn DS Mecham RP Barondes SH. The Elastin Receptor: A Galactoside-Binding Protein. Science (1988) 239:1539–41. doi: 10.1126/science.2832941 2832941

[31] Mecham RP Hinek A Entwistle R Wrenn DS Griffin GL Senior RM. Elastin Binds to a Multifunctional 67-Kilodalton Peripheral Membrane Protein. Biochemistry (1989) 28:3716–22. doi: 10.1021/bi00435a014 2546580

[32] Bonten E van der Spoel A Fornerod M Grosveld G d’Azzo A. Characterization of Human Lysosomal Neuraminidase Defines the Molecular Basis of the Metabolic Storage Disorder Sialidosis. Genes Dev (1996) 10:3156–69. doi: 10.1101/gad.10.24.3156 8985184

[33] van der Spoel A Bonten E d’Azzo A. Transport of Human Lysosomal Neuraminidase to Mature Lysosomes Requires Protective Protein/Cathepsin A. EMBO J (1998) 17:1588–97. doi: 10.1093/emboj/17.6.1588 PMC1170506 9501080

[34] Hoogeveen AT Verheijen FW Galjaard H. The Relation Between Human Lysosomal Beta-Galactosidase and its Protective Protein. J Biol Chem (1983) 258:12143–6. doi: 10.1016/S0021-9258(17)44147-0 6415049

[35] Morreau H Galjart NJ Willemsen R Gillemans N Zhou XY d’Azzo A. Human Lysosomal Protective Protein. Glycosylation, Intracellular Transport, and Association With Beta-Galactosidase in the Endoplasmic Reticulum. J Biol Chem (1992) 267:17949–56. doi: 10.1016/S0021-9258(19)37135-2 1387645

[36] Galjart NJ Morreau H Willemsen R Gillemans N Bonten EJ d’Azzo A. Human Lysosomal Protective Protein has Cathepsin a-Like Activity Distinct From its Protective Function. J Biol Chem (1991) 266:14754–62. doi: 10.1016/S0021-9258(18)98751-X 1907282

[37] Malvagia S Morrone A Caciotti A Bardelli T d’Azzo A Ancora G. New Mutations in the PPBG Gene Lead to Loss of PPCA Protein Which Affects the Level of the Beta-Galactosidase/Neuraminidase Complex and the EBP-Receptor. Mol Genet Metab (2004) 82:48–55. doi: 10.1016/j.ymgme.2004.02.007 15110321

[38] Duca L Blanchevoye C Cantarelli B Ghoneim C Dedieu S Delacoux F. The Elastin Receptor Complex Transduces Signals Through the Catalytic Activity of its Neu-1 Subunit. J Biol Chem (2007) 282:12484–91. doi: 10.1074/jbc.M609505200 17327233

[39] d’Azzo A Bonten E. Molecular Mechanisms of Pathogenesis in a Glycosphingolipid and a Glycoprotein Storage Disease. Biochem Soc Trans (2010) 38:1453–7. doi: 10.1042/BST0381453 PMC3129614 21118106

[40] Uemura T Shiozaki K Yamaguchi K Miyazaki S Satomi S Kato K. Contribution of Sialidase NEU1 to Suppression of Metastasis of Human Colon Cancer Cells Through Desialylation of Integrin Beta4. Oncogene (2009) 28:1218–29. doi: 10.1038/onc.2008.471 19151752

[41] Jayanth P Amith SR Gee K Szewczuk MR. Neu1 Sialidase and Matrix Metalloproteinase-9 Cross-Talk is Essential for Neurotrophin Activation of Trk Receptors and Cellular Signaling. Cell Signal (2010) 22:1193–205. doi: 10.1016/j.cellsig.2010.03.011 20347965

[42] Lillehoj EP Hyun SW Feng C Zhang L Liu A Guang W. NEU1 Sialidase Expressed in Human Airway Epithelia Regulates Epidermal Growth Factor Receptor (EGFR) and MUC1 Protein Signaling. J Biol Chem (2012) 287:8214–31. doi: 10.1074/jbc.M111.292888 PMC3318723 22247545

[43] Amith SR Jayanth P Franchuk S Finlay T Seyrantepe V Beyaert R. Neu1 Desialylation of Sialyl Alpha-2,3-Linked Beta-Galactosyl Residues of TOLL-Like Receptor 4 is Essential for Receptor Activation and Cellular Signaling. Cell Signal (2010) 22:314–24. doi: 10.1016/j.cellsig.2009.09.038 19796680

[44] Amith SR Jayanth P Franchuk S Siddiqui S Seyrantepe V Gee K. Dependence of Pathogen Molecule-Induced Toll-Like Receptor Activation and Cell Function on Neu1 Sialidase. Glycoconj J (2009) 26:1197–212. doi: 10.1007/s10719-009-9239-8 19430901

[45] Jansen AJ Josefsson EC Rumjantseva V Liu QP Falet H Bergmeier W. Desialylation Accelerates Platelet Clearance After Refrigeration and Initiates Gpibalpha Metalloproteinase-Mediated Cleavage in Mice. Blood (2012) 119:1263–73. doi: 10.1182/blood-2011-05-355628 PMC3277358 22101895

[46] Rusciani A Duca L Sartelet H Chatron-Colliet A Bobichon H Ploton D. Elastin Peptides Signaling Relies on Neuraminidase-1-Dependent Lactosylceramide Generation. PloS One (2010) 5:e14010. doi: 10.1371/journal.pone.0014010 21103358 PMC2982818

[47] Chavas LM Tringali C Fusi P Venerando B Tettamanti G Kato R. Crystal Structure of the Human Cytosolic Sialidase Neu2. Evidence for the Dynamic Nature of Substrate Recognition. J Biol Chem (2005) 280:469–75. doi: 10.1074/jbc.M411506200 15501818

[48] Magesh S Suzuki T Miyagi T Ishida H Kiso M. Homology Modeling of Human Sialidase Enzymes NEU1, NEU3 and NEU4 Based on the Crystal Structure of NEU2: Hints for the Design of Selective NEU3 Inhibitors. J Mol Graph Model (2006) 25:196–207. doi: 10.1016/j.jmgm.2005.12.006 16427342

[49] Maurice P Baud S Bocharova OV Bocharov EV Kuznetsov AS Kawecki C. New Insights Into Molecular Organization of Human Neuraminidase-1: Transmembrane Topology and Dimerization Ability. Sci Rep (2016) 6:38363. doi: 10.1038/srep38363 27917893 PMC5137157

[50] Gorelik A Illes K Hasan SMN Nagar B Mazhab-Jafari MT. Structure of the Murine Lysosomal Multienzyme Complex Core. Sci Adv (2021) 7:eabf4155. doi: 10.1126/sciadv.abf4155 33980489 PMC8115914

[51] Blood CH Sasse J Brodt P Zetter BR. Identification of a Tumor Cell Receptor for VGVAPG, an Elastin-Derived Chemotactic Peptide. J Cell Biol (1988) 107:1987–93. doi: 10.1083/jcb.107.5.1987 PMC2115331 2846590

[52] Senior RM Griffin GL Mecham RP Wrenn DS Prasad KU Urry DW. Val-Gly-Val-Ala-Pro-Gly, a Repeating Peptide in Elastin, is Chemotactic for Fibroblasts and Monocytes. J Cell Biol (1984) 99:870–4. doi: 10.1083/jcb.99.3.870 PMC2113419 6547961

[53] Chatron-Colliet A Lalun N Terryn C Kurdykowski S Lorenzato M Rusciani A. The Elastin Peptide (VGVAPG)3 Induces the 3D Reorganisation of PML-Nbs and SC35 Speckles Architecture, and Accelerates Proliferation of Fibroblasts and Melanoma Cells. Histochem Cell Biol (2015) 143:245–58. doi: 10.1007/s00418-014-1274-2 25274422

[54] Ghuysen-Itard AF Robert L Jacob MP. Effect of Elastin Peptides on Cell Proliferation. C R Acad Sci III (1992) 315:473–8. 1297524

[55] Mochizuki S Brassart B Hinek A. Signaling Pathways Transduced Through the Elastin Receptor Facilitate Proliferation of Arterial Smooth Muscle Cells. J Biol Chem (2002) 277:44854–63. doi: 10.1074/jbc.M205630200 12244048

[56] Tajima S Wachi H Uemura Y Okamoto K. Modulation by Elastin Peptide VGVAPG of Cell Proliferation and Elastin Expression in Human Skin Fibroblasts. Arch Dermatol Res (1997) 289:489–92. doi: 10.1007/s004030050227 9266029

[57] Fahem A Robinet A Cauchard JH Duca L Soula-Rothhut M Rothhut B. Elastokine-Mediated Up-Regulation of MT1-MMP is Triggered by Nitric Oxide in Endothelial Cells. Int J Biochem Cell Biol (2008) 40:1581–96. doi: 10.1016/j.biocel.2007.11.022 18206415

[58] Huet E Brassart B Cauchard JH Debelle L Birembaut P Wallach J. Cumulative Influence of Elastin Peptides and Plasminogen on Matrix Metalloproteinase Activation and Type I Collagen Invasion by HT-1080 Fibrosarcoma Cells. Clin Exp Metastasis (2002) 19:107–17. doi: 10.1023/A:1014547324918 11964074

[59] Robinet A Fahem A Cauchard JH Huet E Vincent L Lorimier S. Elastin-Derived Peptides Enhance Angiogenesis by Promoting Endothelial Cell Migration and Tubulogenesis Through Upregulation of MT1-MMP. J Cell Sci (2005) 118:343–56. doi: 10.1242/jcs.01613 15632106

[60] Faury G Usson Y Robert-Nicoud M Robert L Verdetti J. Nuclear and Cytoplasmic Free Calcium Level Changes Induced by Elastin Peptides in Human Endothelial Cells. Proc Natl Acad Sci USA (1998) 95:2967–72. doi: 10.1073/pnas.95.6.2967 PMC19678 9501199

[61] Jacob MP Fulop T Jr Foris G Robert L. Effect of Elastin Peptides on Ion Fluxes in Mononuclear Cells, Fibroblasts, and Smooth Muscle Cells. Proc Natl Acad Sci USA (1987) 84:995–9. doi: 10.1073/pnas.84.4.995 PMC304348 3103130

[62] Ntayi C Labrousse AL Debret R Birembaut P Bellon G Antonicelli F. Elastin-Derived Peptides Upregulate Matrix Metalloproteinase-2-Mediated Melanoma Cell Invasion Through Elastin-Binding Protein. J Invest Dermatol (2004) 122:256–65. doi: 10.1046/j.0022-202X.2004.22228.x 15009703

[63] Baranek T Debret R Antonicelli F Lamkhioued B Belaaouaj A Hornebeck W. Elastin Receptor (Spliced Galactosidase) Occupancy by Elastin Peptides Counteracts Proinflammatory Cytokine Expression in Lipopolysaccharide-Stimulated Human Monocytes Through NF-Kappab Down-Regulation. J Immunol (2007) 179:6184–92. doi: 10.4049/jimmunol.179.9.6184 17947694

[64] Fulop T Khalil A Larbi A. The Role of Elastin Peptides in Modulating the Immune Response in Aging and Age-Related Diseases. Pathol Biol (Paris) (2012) 60:28–33. doi: 10.1016/j.patbio.2011.10.006 22099332

[65] Robinet A Millart H Oszust F Hornebeck W Bellon G. Binding of Elastin Peptides to s-Gal Protects the Heart Against Ischemia/Reperfusion Injury by Triggering the RISK Pathway. FASEB J (2007) 21:1968–78. doi: 10.1096/fj.06-6477com 17341689

[66] Antonicelli F Bellon G Lorimier S Hornebeck W. Role of the Elastin Receptor Complex (s-Gal/Cath-a/Neu-1) in Skin Repair and Regeneration. Wound Repair Regener (2009) 17:631–8. doi: 10.1111/j.1524-475X.2009.00525.x 19769716

[67] Attia-Vigneau J Terryn C Lorimier S Sandre J Antonicelli F Hornebeck W. Regeneration of Human Dermis by a Multi-Headed Peptide. J Invest Dermatol (2014) 134:58–67. doi: 10.1038/jid.2013.290 23812301

[68] Varga Z Jacob MP Robert L Fulop T Jr. Identification and Signal Transduction Mechanism of Elastin Peptide Receptor in Human Leukocytes. FEBS Lett (1989) 258:5–8. doi: 10.1016/0014-5793(89)81602-3 2556298

[69] Duca L Debelle L Debret R Antonicelli F Hornebeck W Haye B. the Elastin Peptides-Mediated Induction of Pro-Collagenase-1 Production by Human Fibroblasts Involves Activation of MEK/ERK Pathway *via* PKA- and PI(3)K-Dependent Signaling. FEBS Lett (2002) 524:193–8. doi: 10.1016/S0014-5793(02)03057-0 12135766

[70] Duca L Lambert E Debret R Rothhut B Blanchevoye C Delacoux F. Elastin Peptides Activate Extracellular Signal-Regulated Kinase 1/2 *via* a Ras-Independent Mechanism Requiring Both P110gamma/Raf-1 and Protein Kinase a/B-Raf Signaling in Human Skin Fibroblasts. Mol Pharmacol (2005) 67:1315–24. doi: 10.1124/mol.104.002725 15653554

[71] Wrenn DS Griffin GL Senior RM Mecham RP. Characterization of Biologically Active Domains on Elastin: Identification of a Monoclonal Antibody to a Cell Recognition Site. Biochemistry (1986) 25:5172–6. doi: 10.1021/bi00366a028 2429696

[72] Dale MA Xiong W Carson JS Suh MK Karpisek AD Meisinger TM. Elastin-Derived Peptides Promote Abdominal Aortic Aneurysm Formation by Modulating M1/M2 Macrophage Polarization. J Immunol (2016) 196:4536–43. doi: 10.4049/jimmunol.1502454 PMC4880455 27183603

[73] Guo G Gehle P Doelken S Martin-Ventura JL von Kodolitsch Y Hetzer R. Induction of Macrophage Chemotaxis by Aortic Extracts From Patients With Marfan Syndrome is Related to Elastin Binding Protein. PloS One (2011) 6:e20138. doi: 10.1371/journal.pone.0020138 21647416 PMC3103536

[74] Guo G Munoz-Garcia B Ott CE Grunhagen J Mousa SA Pletschacher A. Antagonism of Gxxpg Fragments Ameliorates Manifestations of Aortic Disease in Marfan Syndrome Mice. Hum Mol Genet (2013) 22:433–43. doi: 10.1093/hmg/dds439 23100322

[75] Hance KA Tataria M Ziporin SJ Lee JK Thompson RW. Monocyte Chemotactic Activity in Human Abdominal Aortic Aneurysms: Role of Elastin Degradation Peptides and the 67-Kd Cell Surface Elastin Receptor. J Vasc Surg (2002) 35:254–61. doi: 10.1067/mva.2002.120382 11854722

[76] Houghton AM Quintero PA Perkins DL Kobayashi DK Kelley DG Marconcini LA. Elastin Fragments Drive Disease Progression in a Murine Model of Emphysema. J Clin Invest (2006) 116:753–9. doi: 10.1172/JCI25617 PMC1361346 16470245

[77] Coquerel B Poyer F Torossian F Dulong V Bellon G Dubus I. Elastin-Derived Peptides: Matrikines Critical for Glioblastoma Cell Aggressiveness in a 3-D System. Glia (2009) 57:1716–26. doi: 10.1002/glia.20884 19373935

[78] Kawecki C Bocquet O Schmelzer CEH Heinz A Ihling C Wahart A. Identification of CD36 as a New Interaction Partner of Membrane NEU1: Potential Implication in the Pro-Atherogenic Effects of the Elastin Receptor Complex. Cell Mol Life Sci (2019) 76:791–807. doi: 10.1007/s00018-018-2978-6 30498996 PMC6514072

[79] Guo G Booms P Halushka M Dietz HC Ney A Stricker S. Induction of Macrophage Chemotaxis by Aortic Extracts of the Mgr Marfan Mouse Model and a Gxxpg-Containing Fibrillin-1 Fragment. Circulation (2006) 114:1855–62. doi: 10.1161/CIRCULATIONAHA.105.601674 17030689

[80] Hata K Koseki K Yamaguchi K Moriya S Suzuki Y Yingsakmongkon S. Limited Inhibitory Effects of Oseltamivir and Zanamivir on Human Sialidases. Antimicrob Agents Chemother (2008) 52:3484–91. doi: 10.1128/AAC.00344-08 PMC2565904 18694948

[81] Richards MR Guo T Hunter CD Cairo CW. Molecular Dynamics Simulations of Viral Neuraminidase Inhibitors With the Human Neuraminidase Enzymes: Insights Into Isoenzyme Selectivity. Bioorg Med Chem (2018) 26:5349–58. doi: 10.1016/j.bmc.2018.05.035 29903413

[82] Burmeister WP Henrissat B Bosso C Cusack S Ruigrok RW. Influenza B Virus Neuraminidase can Synthesize its Own Inhibitor. Structure (1993) 1:19–26. doi: 10.1016/0969-2126(93)90005-2 8069621

[83] Varghese JN Smith PW Sollis SL Blick TJ Sahasrabudhe A McKimm-Breschkin JL. Drug Design Against a Shifting Target: A Structural Basis for Resistance to Inhibitors in a Variant of Influenza Virus Neuraminidase. Structure (1998) 6:735–46. doi: 10.1016/S0969-2126(98)00075-6 9655825

[84] Varghese JN McKimm-Breschkin JL Caldwell JB Kortt AA Colman PM. the Structure of the Complex Between Influenza Virus Neuraminidase and Sialic Acid, the Viral Receptor. Proteins (1992) 14:327–32. doi: 10.1002/prot.340140302 1438172

[85] Magesh S Moriya S Suzuki T Miyagi T Ishida H Kiso M. Design, Synthesis, and Biological Evaluation of Human Sialidase Inhibitors. Part 1: Selective Inhibitors of Lysosomal Sialidase (NEU1). Bioorg Med Chem Lett (2008) 18:532–7. doi: 10.1016/j.bmcl.2007.11.084 18068975

[86] Guo T Heon-Roberts R Zou C Zheng R Pshezhetsky AV Cairo CW. Selective Inhibitors of Human Neuraminidase 1 (NEU1). J Med Chem (2018) 61:11261–79. doi: 10.1021/acs.jmedchem.8b01411 30457869

[87] Hyun SW Liu A Liu Z Cross AS Verceles AC Magesh S. The NEU1-Selective Sialidase Inhibitor, C9-Butyl-Amide-DANA, Blocks Sialidase Activity and NEU1-Mediated Bioactivities in Human Lung *In Vitro* and Murine Lung *In Vivo* . Glycobiology (2016) 26:834–49. doi: 10.1093/glycob/cww060 PMC5884327 27226251

[88] Luzina IG Lillehoj EP Lockatell V Hyun SW Lugkey KN Imamura A. Therapeutic Effect of Neuraminidase-1-Selective Inhibition in Mouse Models of Bleomycin-Induced Pulmonary Inflammation and Fibrosis. J Pharmacol Exp Ther (2021) 376:136–46. doi: 10.1124/jpet.120.000223 PMC7788353 33139318

[89] Albrecht C Kuznetsov AS Appert-Collin A Dhaideh Z Callewaert M Bershatsky YV. Transmembrane Peptides as a New Strategy to Inhibit Neuraminidase-1 Activation. Front Cell Dev Biol (2020) 8:611121. doi: 10.3389/fcell.2020.611121 33392200 PMC7772355

[90] Albrecht C Akissi ZLE Yao-Kouassi PA Alabdul Magid A Maurice P Duca L. Identification and Evaluation of New Potential Inhibitors of Human Neuraminidase 1 Extracted From Olyra Latifolia L.: A Preliminary Study. Biomedicines (2021) 9:411. doi: 10.3390/biomedicines9040411 33920466 PMC8070403

[91] Park S Kim N Nhiem NX Kwak HJ Yang HW Kim SH. Neuraminidase Inhibitors From the Roots of Caragana Sinica. Chem Biodivers (2020) 17:e2000470. doi: 10.1002/cbdv.202000470 32996697

[92] Yoo G Park JH Kim SH. Neuraminidase Inhibitory Diarylheptanoids From Alpinia Officinarum: *In Vitro* and Molecular Docking Studies. Bioorg Chem (2021) 107:104526. doi: 10.1016/j.bioorg.2020.104526 33309269

[93] Lee C Liu A Miranda-Ribera A Hyun SW Lillehoj EP Cross AS. NEU1 Sialidase Regulates the Sialylation State of CD31 and Disrupts CD31-Driven Capillary-Like Tube Formation in Human Lung Microvascular Endothelia. J Biol Chem (2014) 289:9121–35. doi: 10.1074/jbc.M114.555888 PMC3979388 24550400

[94] Hinek A Bodnaruk TD Bunda S Wang Y Liu K. Neuraminidase-1, a Subunit of the Cell Surface Elastin Receptor, Desialylates and Functionally Inactivates Adjacent Receptors Interacting With the Mitogenic Growth Factors PDGF-BB and IGF-2. Am J Pathol (2008) 173:1042–56. doi: 10.2353/ajpath.2008.071081 PMC2543072 18772331

[95] Crudden C Shibano T Song D Suleymanova N Girnita A Girnita L. Blurring Boundaries: Receptor Tyrosine Kinases as Functional G Protein-Coupled Receptors. Int Rev Cell Mol Biol (2018) 339:1–40. doi: 10.1016/bs.ircmb.2018.02.006 29776602

[96] Kilpatrick LE Hill SJ. Transactivation of G Protein-Coupled Receptors (Gpcrs) and Receptor Tyrosine Kinases (Rtks): Recent Insights Using Luminescence and Fluorescence Technologies. Curr Opin Endocr Metab Res (2021) 16:102–12. doi: 10.1016/j.coemr.2020.10.003 PMC7960640 33748531

[97] Wang W Qiao Y Li Z. New Insights Into Modes of GPCR Activation. Trends Pharmacol Sci (2018) 39:367–86. doi: 10.1016/j.tips.2018.01.001 29395118

[98] Mira E Lacalle RA Gonzalez MA Gomez-Mouton C Abad JL Bernad A. A Role for Chemokine Receptor Transactivation in Growth Factor Signaling. EMBO Rep (2001) 2:151–6. doi: 10.1093/embo-reports/kve027 PMC1083823 11258708

[99] Delcourt N Bockaert J Marin P. GPCR-Jacking: From a New Route in RTK Signalling to a New Concept in GPCR Activation. Trends Pharmacol Sci (2007) 28:602–7. doi: 10.1016/j.tips.2007.09.007 18001849

[100] Cattaneo F Guerra G Parisi M De Marinis M Tafuri D Cinelli M. Cell-Surface Receptors Transactivation Mediated by G Protein-Coupled Receptors. Int J Mol Sci (2014) 15:19700–28. doi: 10.3390/ijms151119700 PMC4264134 25356505

[101] Abdulkhalek S Amith SR Franchuk SL Jayanth P Guo M Finlay T. Neu1 Sialidase and Matrix Metalloproteinase-9 Cross-Talk is Essential for Toll-Like Receptor Activation and Cellular Signaling. J Biol Chem (2011) 286:36532–49. doi: 10.1074/jbc.M111.237578 PMC3196117 21873432

[102] Abdulkhalek S Guo M Amith SR Jayanth P Szewczuk MR. G-Protein Coupled Receptor Agonists Mediate Neu1 Sialidase and Matrix Metalloproteinase-9 Cross-Talk to Induce Transactivation of TOLL-Like Receptors and Cellular Signaling. Cell Signal (2012) 24:2035–42. doi: 10.1016/j.cellsig.2012.06.016 22759791

[103] Abdulkhalek S Szewczuk MR. Neu1 Sialidase and Matrix Metalloproteinase-9 Cross-Talk Regulates Nucleic Acid-Induced Endosomal TOLL-Like Receptor-7 and -9 Activation, Cellular Signaling and Pro-Inflammatory Responses. Cell Signal (2013) 25:2093–105. doi: 10.1016/j.cellsig.2013.06.010 23827939

[104] Alghamdi F Guo M Abdulkhalek S Crawford N Amith SR Szewczuk MR. A Novel Insulin Receptor-Signaling Platform and its Link to Insulin Resistance and Type 2 Diabetes. Cell Signal (2014) 26:1355–68. doi: 10.1016/j.cellsig.2014.02.015 24583283

[105] Gilmour AM Abdulkhalek S Cheng TS Alghamdi F Jayanth P O’Shea LK. A Novel Epidermal Growth Factor Receptor-Signaling Platform and its Targeted Translation in Pancreatic Cancer. Cell Signal (2013) 25:2587–603. doi: 10.1016/j.cellsig.2013.08.008 23993964

[106] Haxho F Haq S Szewczuk MR. Biased G Protein-Coupled Receptor Agonism Mediates Neu1 Sialidase and Matrix Metalloproteinase-9 Crosstalk to Induce Transactivation of Insulin Receptor Signaling. Cell Signal (2018) 43:71–84. doi: 10.1016/j.cellsig.2017.12.006 29277445

[107] Kozel BA Mecham RP. Elastic Fiber Ultrastructure and Assembly. Matrix Biol (2019) 84:31–40. doi: 10.1016/j.matbio.2019.10.002 31669522 PMC8409341

[108] Ozsvar J Yang C Cain SA Baldock C Tarakanova A Weiss AS. Tropoelastin and Elastin Assembly. Front Bioeng Biotechnol (2021) 9:643110. doi: 10.3389/fbioe.2021.643110 33718344 PMC7947355

[109] Hauser AS Attwood MM Rask-Andersen M Schioth HB Gloriam DE. Trends in GPCR Drug Discovery: New Agents, Targets and Indications. Nat Rev Drug Discov (2017) 16:829–42. doi: 10.1038/nrd.2017.178 PMC6882681 29075003

[110] Snider J Kittanakom S Damjanovic D Curak J Wong V Stagljar I. Detecting Interactions With Membrane Proteins Using a Membrane Two-Hybrid Assay in Yeast. Nat Protoc (2010) 5:1281–93. doi: 10.1038/nprot.2010.83 20595957

[111] Rudenko G Bonten E d’Azzo A Hol WG. Three-Dimensional Structure of the Human ‘Protective Protein’: Structure of the Precursor Form Suggests a Complex Activation Mechanism. Structure (1995) 3:1249–59. doi: 10.1016/S0969-2126(01)00260-X 8591035

[112] Achyuthan KE Achyuthan AM. Comparative Enzymology, Biochemistry and Pathophysiology of Human Exo-Alpha-Sialidases (Neuraminidases). Comp Biochem Physiol B Biochem Mol Biol (2001) 129:29–64. doi: 10.1016/S1096-4959(01)00372-4 11337249

